# Calyculins and Related Marine Natural Products as Serine-Threonine Protein Phosphatase PP1 and PP2A Inhibitors and Total Syntheses of Calyculin A, B, and C

**DOI:** 10.3390/md80100122

**Published:** 2010-01-21

**Authors:** Annika E. Fagerholm, Damien Habrant, Ari M. P. Koskinen

**Affiliations:** Laboratory of Organic Chemistry, Helsinki University of Technology, PO Box 6100, FIN-02015 HUT, Finland; E-Mails: afagerho@cc.hut.fi (A.E.F.); habrant@cc.hut.fi (D.H.)

**Keywords:** marine natural products, total synthesis, protein phosphatase inhibitors

## Abstract

Calyculins, highly cytotoxic polyketides, originally isolated from the marine sponge *Discodermia calyx* by Fusetani and co-workers, belong to the lithistid sponges group. These molecules have become interesting targets for cell biologists and synthetic organic chemists. The serine/threonine protein phosphatases play an essential role in the cellular signalling, metabolism, and cell cycle control. Calyculins express potent protein phosphatase 1 and 2A inhibitory activity, and have therefore become valuable tools for cellular biologists studying intracellular processes and their control by reversible phosphorylation. Calyculins might also play an important role in the development of several diseases such as cancer, neurodegenerative diseases, and type 2-*diabetes mellitus*. The fascinating structures of calyculins have inspired various groups of synthetic organic chemists to develop total syntheses of the most abundant calyculins A and C. However, with fifteen chiral centres, a cyano-capped tetraene unit, a phosphate-bearing spiroketal, an *anti, anti, anti* dipropionate segment, an α-chiral oxazole, and a trihydroxylated γ-amino acid, calyculins reach versatility that only few natural products can surpass, and truly challenge modern chemists’ asymmetric synthesis skills.

## 1. Introduction

Nature offers an endless source of inspiration to synthetic organic chemists. Because water covers more than 70% of Earth’s surface, it is only logical that the number of new molecules isolated from marine species is enormous, including structures that have never been found from terrestial organisms. This interest has led to the discovery of many compounds with very promising biological activity [1]. Among these compounds is the calyculin family. Various studies have shown that calyculins are potent inhibitors of protein phosphatases 1 and 2A, opening up numerous possibilities for their therapeutic use [2–7].

**Figure 1:**
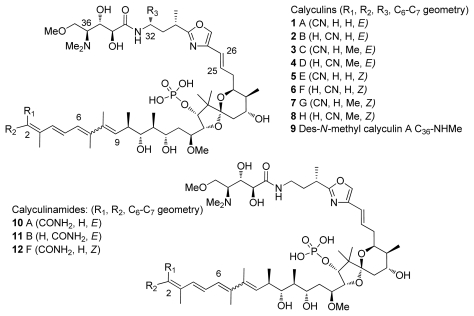
Calyculins and calyculinamides.

Calyculins are a class of highly cytotoxic metabolites originally isolated from the marine sponge *Discodermia calyx* by Fusetani and co-workers. The first member, calyculin A, was isolated in 1986 from a sponge collected in the Gulf of Sagami, near Tokyo Bay [8–15]. The sponge still remains the primary source of the natural product. The structures of different calyculins and structurally-related calyculinamides are shown in Figure 1. The most naturally abundant members of the family are calyculins A and C.

The structure of complex natural products may sometimes lead, even with the help of modern analytical methods, to misassignments of the absolute stereochemistry. In such cases, total synthesis can be the key for proving the absolute stereochemistry of the natural product. Calyculins provide an excellent example as Shioiri and co-workers ascertained the absolute stereochemistry of calyculins by synthesis in 1991 shortly after Fusetani disclosed the absolute configuration of calyculin A [16,17]. In their original article, Fusetani and co-workers presented a structure for calyculin A that appeared to be the enantiomer of the natural product [11]. Although being very clear about the uncertainty of the absolute configuration, the then ongoing synthetic efforts towards the calyculins had been directed to the non-natural enantiomer. As a consequence, three of the six published total syntheses of calyculins have yielded the wrong enantiomer [18–23].

## 2. Importance of Protein Phosphatases

Phosphorylation-dephosphorylation of proteins is one of the most essential mechanisms for the proper functioning of cells. It affects almost all cellular functions such as metabolism, signal transduction, cell division, and memory. Protein kinases have long been known for the regulatory properties of phosphorylation and dephosphorylation. Although it has been recognised only later, protein phosphatases (PP) have also a great influence for these regulation processes. Phosphatases that catalyze dephosphorylation of serine and threonine residues are encoded by the phospho protein phosphatase (PPP) and protein phosphatase magnesium-dependent (PPM) gene families, whereas the protein tyrosine phosphatases (PTPs) dephosphorylate phosphotyrosine amino acids [2,3]. PP enzymes play a very dynamic role in cellular signalling, particularly because they can be turned on and off through very tight regulation of their subunit composition and selective targeting. These functions are regulated by allosteric modification using second messengers and reversible protein phosphorylation to create specific subcellular multi-protein signalling modules [2,6,7].

The total number of phosphatases discovered is over 100 but it has been estimated that the total number could be as many as 1,000 [6]. PP1, PP2A, PP2B, and PP2C are the most widely studied phosphatases and also account for the majority of the protein serine/threonine activity *in vivo*. PP1, PP2A, and PP2B belong to the family of PPPs and their enzymatic activity is dependent upon Ca^2+^/Calmodulin, whereas PP2C of the PPM family is Mg^2+^ dependent [2,3].

Extracellular signals, such as hormones and growth factors, affect the regulatory subunits and thereby modify the substrate specificity of PP1, which is involved in glycogen metabolism, muscle contraction, cell cycle progression, neuronal activities, and splicing of RNA. Recently, PP2A has been the focus of important interest since it accounts for 1% of total cellular proteins, and for the major portion of serine and threonine phosphatase activity in most tissues and cells. Although PP2A is involved in a great variety of cellular processes, including cell metabolism, signalling, and cell cycle control as well as the control of telomerase activity, its specific role is less delineated [2,3].

The holoenzyme of PP2A consists of three subunits, named A, B, and C. The catalytic subunit C is always associated with the scaffolding subunit A, which modulates its enzymatic properties by coordinating the protein-protein targeting to protein kinases and cytoskeletal proteins [7]. The holoenzyme of PP1 contains also a catalytic subunit C. PP1’s and PP2A’s C subunits are structurally related, and share 50% amino acid identity [6]. The regulative subunit B, subdivided into B, B′, B″, is encoded by separate genes, and can bind to AC with wide variety of heteromeric complexes. It is believed that individual subunits cannot exist individually *in vivo*; however, AC dimers are abundant in tissues. To date, two isoforms (α, β) of subunits A and C have been described, and there is an ever-growing number of B-type isoforms. The homologues of mammalian PP2A subunits have been identified from diverse origins such as algae, higher plants, and yeast. Moreover, although PP2A is primarily a serine and threonine phosphatase, it can, in specific circumstances, display an independent phosphotyrosyl phosphatase (PTP) activity. The diversity and selectivity of PP2A has been linked to the coverable three dimensional holoenzyme [7].

Protein phosphatase signalling plays an important role in many human diseases [3–5]. Unfortunately, studies towards determining the signalling mechanism have been slowed down by the absence of a PP2A crystal structure [4]. Many observations support the role of PP2A in tumorogenesis although PP2A inhibitors can also display anti-tumour activity [3–5]. The mutations in the gene encoding the subunit A in human breast, lung, and colorectal carcinomas, as well as in melanomas strengthen the notion of tumorigenesis activity [4,7]. However, it has not been unequivocally established so far whether such mutations, examples of which have been found in human cancer cells, result in the activation of an oncogenic function or rather in the inactivation of the presumed tumour suppressive role of PP2A. The exact effect of PP2As has been found complicated since it can exert inhibiting as well as stimulating control on cell proliferation. This might indicate activity of several different PP2A complexes during these processes [5].

The major members of PPP family are highly concentrated in the brain, and are fundamental elements of complex signalling system controlling neuronal function. PP1 is widely distributed in neurons and has multiple functions. Targeted inhibition of PP1 is a potential strategy for minimizing the symptoms associated with Parkinson’s disease [4]. PP2A activity also affects human neurodegenerative diseases. In Alzheimer’s disease, the activity levels of PP2A are significantly decreased. Altogether, PP2A-dependent PI 3-kinase signalling plays a crucial role in neuronal survival [4,7].

Both PP1 and PP2A are involved in the mediation of insulin action on carbohydrate and lipid metabolism. More specifically, activation of PP1 and inactivation of PP2A can affect insulin stimulation. Type 2 *diabetes mellitus* is characterized by variation of insulin resistance. Therefore, molecules involved in the insulin signalling cascade are potential targets for therapeutic drug design; both PP1 and PP2A have been involved in these studies.

PP2A signalling also regulates the transcription factors Sp1 and NK-κB which are essential modulators of cellular gene expression and viral transcription of many human viruses, such as HIV-1, cytomegalovirus, hepatitis B, herpes simplex type 1, Epstein-Bass virus, and papillomavirus. Recent studies also suggest that PP2A signalling participates in parasite-transmitted human diseases such as malaria [7].

## 3. Inhibition of Protein Phosphatases PP1 and PP2A by Naturally Occurring Toxins

In contrast to many enzymes, protein phosphatases, especially PP1 and PP2A, exhibit broad and overlapping substrate specificity, with no apparent substrate consensus sequence. Because the protein phosphatases affect other proteins and have literally hundred of substrates, it has been challenging to describe the mode of action of these biological catalysts and their regulation. For that reason, much of the information gathered from the functioning of protein phosphatases is based on inhibition studies [6].

Protein inhibitors have been used to study the mechanism of protein phosphatase inhibition. However, they suffer from some shortages: proteolytic degradation, poor membrane permeability, high molecular weight, potential instability, and often unavailability in sufficient quantity. To avoid these problems, small molecule inhibitors are often used. Many naturally occurring molecules, with wide structurally diversity, have been identified to either selectively or specifically inhibit the phosphatases. Alkaloids, terpenes, oligosaccharides, and polyketides have evolved to imitate and/or complement small areas of molecular surfaces of protein-peptides [6].

**Figure 2:**
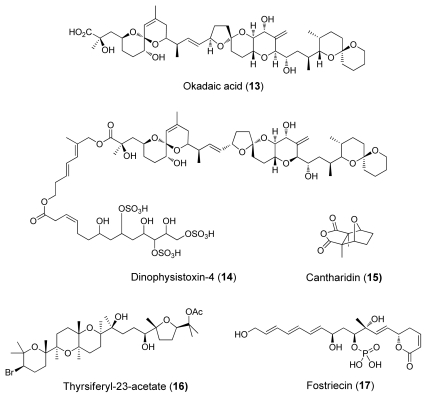
PP2A-selective inhibitors.

**Figure 3:**
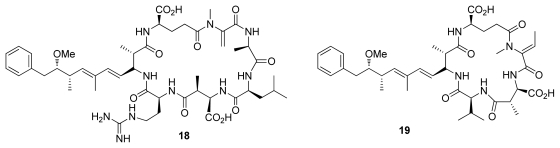
Right: microcystin-LR (**18**); left: motuporin (**19**).

**Figure 4:**
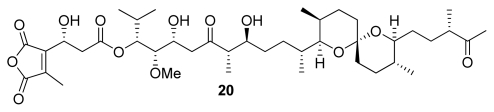
Tautomycin **20**.

Several natural products from different structural groups have been identified to inhibit serine/threonine-specific protein phosphatases. The natural toxin inhibitors are also known as the *okadaic acid class* inhibitors (Figures 1–4). Okadaic acid, the causative agent of diarrhetic seafood poisoning [4], was the first of these inhibitors discovered in 1981. It is a marine polyketide initially found from marine sponges *Halicondria okadai* and *Halicondria melanodocia*.

Cyclic peptides such as microcystins (e.g., microcystin–LR (**18**)) and nodularins were initially isolated from blue green algae and are potent inhibitors of PP1 and PP2A, but poor PP2B and PP2C inhibitors. Another cyclic peptide motuporin (**19**), also known as nodularin-V, was isolated from the marine sponge *Theonella swinhoei gray* (Figure 3) [4]. Both microcystins and motuporin share the rare aminoacid ADDA, which interacts with the hydrophobic groove of PP1.

As mentioned earlier several different structural groups can bind PP1 and PP2A, which makes the classification of these inhibitors challenging. The inhibitors can be classified by structure but a valid choice is classification based on PP1/PP2A selectivity. Based on different inhibition studies and screening, a large number of structurally interesting natural products has been identified to bind PP1 and PP2A more or less selectively. The IC_50_ values of selected molecules are collected in Table 1, as well as the origin and structural class.

From the biological activity data (Table 1) it can be observed that okadaic acid (**13**), dinophysistoxin (**14**), cantharidin (**15**) and its derivative, thyrsiferyl-23-acetate (**16**), as well as phosphate-bearing inhibitor fostriecin (**17**) (Figure 2) are selective PP2A inhibitors. Although some common structures such as spiroketal moieties can be identified, the observed PP1 and PP2A differences cannot be adequately explained with the current structure-activity relationship data [6]. However, the binding data of Table 1 indicates that tautomycin (**20**) (Figure 4) and calyculins show slight PP1 selectivity. Tautomycin was first isolated from *Streptomyces spiroverticillatus* and is the first inhibitor to display preferential inhibition of PP1 (Figure 4) [4].

Mutagenesis and natural products studies indicate that acidic groove residues are a key feature in the active site of PP1 [4]. This could mean that the binding region in PP2A is more hydrophobic than the one in PP1, and therefore more accessible to hydrophobic inhibitors such as thyrsiferyl-23-acetate (**16**) (Figure 2).

## 4. Calyculins and Related Structures

### 4.1. Origin

The first isolated calyculin was calyculin A (**1**) in 1986, followed by calyculins B-D (**2**–**4**) in 1988, calyculins E-H (**5**–**8**) in 1990 and calyculin J (**25**) in 1997 [9–15]. *D. calyx* belongs to the order of lithistid sponges, which are an artificial assemblage of species of diverse origin known from their ability to produce diverse array of biologically active metabolites such as polyketides, cyclic peptides, alkaloids, pigments, and novel sterols [9].

**Figure 5:**
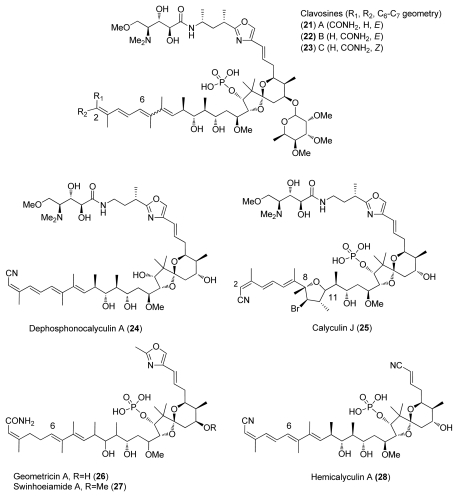
Calyculin related structures.

Calyculin related structures have also been found from other marine sponges, such as *Lamellomorpha strongylata* which was collected at the Chantam Rise off the East Coast of South Island of New Zealand in 1995 and whose extraction afforded calyculinamides A (**10**) and B (**11**) [10]. Calyculinamide A (**10**), calyculinamide F (**12**), des-*N*-methyl calyculin A (**9**), and dephosphocalyculin A (**24**) were isolated in 1997 from *D. calyx* [13]. Further, calyculin derivatives clavosines A-C (**21**–**23**) were isolated in 1998 from the marine sponge *Myriastra clavosa* (Figure 5) [25]. In 2001, Epipolasid sponge *Lufariella geometrica* was collected at Heron Island’s Wistari Reef, Australia, and allowed the isolation of another novel calyculin derivative, geometricin A (**26**) [26]. The latest isolated calyculin derivative is swinhoeiamide A (**27**) from the lithistid sponge *Theonella svinhoei* [27].

To this day, totally eighteen calyculins and calyculin related structures have been isolated. Calyculin A is formed from four different structural regions: C_1_–C_8_ tetraene, C_9_–C_25_ dipropionate spiroketal, C_26_–C_32_ oxazole and C_33_–C_37_ amino acid, these subunits are represented in Figure 1. Calyculins differ from each other by the methyl group at C_32_ and the geometry of C_2,3_ and C_6,7_ olefins [9–15]. The geometry of C_2,3_ and C_6,7_ olefins are also the critical sites of the structural differences in calyculinamides as well as in clavosines. In the latter, the C_21_ hydroxyl group is also in the *S* configuration, and is glycosylated by a trimethoxyrhamnose [10,13,25]. Calyculin J (**25**) is a C_9_ brominated derivative of **1** where C_8–11_ and C_11_ oxygen form a tetrahydrofuran ring. Geometricin A (**26**), swinhoeiamide A (**27**), and hemicalyculin A (**28**) could be described as rump calyculin derivatives since the most significant difference comparing to calyculins is the lack of the polar region [26–28].

### 4.2. Crystal structures of calyculins and their binding to protein phosphatases

Several research groups have studied the structure-activity relationships (SARs) of naturally occurring toxins to PP1 and PP2A. Quinn *et. al.* developed a pharmacophore model for the binding of okadaic acid (**13**), calyculin A (**1**) and microcystin LR (**18**) to PP1 [29]. Competitive binding assays with **13**, **1**, **18** and tautomycin (**20**) suggested that at least these toxins share a common binding site [30]. The absolute stereochemistry of the calyculins was first published in 1991 by Shioiri *et. al*. [16]. The first publication of X-ray structure of PP1 in 1995 [31] soon inspired four docking studies in 1997 [24,32–34]. However, the first two groups, Armstrong and Holmes, used the incorrect enantiomer of crystal structure. Their initial idea was that the binding of the toxins would not change the structure significantly [8]. This seems to be possible for cyclic microcystines and nodularins; however, with open chain molecules such as calyculins, this approach is unlikely [34].

**Figure 6:**
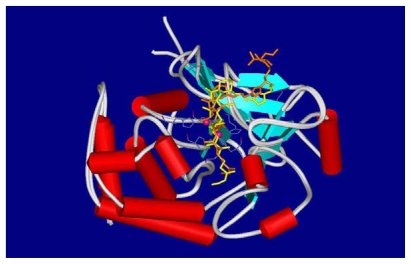
Binding models of calyculin A to PP1.

Calyculins as well as other inhibitors have been targets of continuous study and several binding models have been proposed [28,35–37]. The binding mode of calyculin A (**1**) to the active site of PP1 is shown in Figure 6: **1** is represented according to its crystal structure in orange [35], in yellow is the calyculin model built by Koskinen [34].

The SARs of calyculins indicate that the phosphate, the hydroxyl C_13_, and the hydrophobic polyketide tail are essential for their inhibitory action. The dipeptide portion was less important in the interaction with enzymes, but essential for cytotoxicity [28,35]. However, compared to **1**, dephosphonocalyculin A (**24**) was inactive, which was already examined [15]. This could indicate that phosphate group is less important for the binding.

It should be noted that the published models are still speculative. The prediction of enzyme-inhibitor interaction is challenging because there are so many parameters affecting the system. Site-directed mutagenesis studies and SAR data for serine/threonine protein phosphatases are useful, but the interpretation of the results can be difficult. Even the models of most simple enzymes contain so wide range of contacts that the interpretation is difficult. Calyculin fragments would give a useful addition to PP1, PP2A binding and SAR studies. In future, design of simpler and more selective inhibitors would also be possible.

## 5. Synthetic Approaches towards Calyculins

The fascinating structures of calyculins have drawn a great amount of attention and resources. The first total synthesis of *ent*-calyculin A (**1**) was published by Evans *et al.* in 1992 [18]. Two years later, Masamune *et al.* published the first total synthesis of the natural enantiomer of **1** [19]. In 1996, the Shioiri group published a formal total synthesis of **1** [20]. Total synthesis of *ent*-calyculin A and B by Smith *et al.* [22] and calyculin C by the Armstrong group were published in 1998 [21]. The latest total synthesis of *ent*-calyculin A was published by Barrett *et al.* in 2001 [23]. The Koskinen group has been involved in the preparation of individual fragments [38–46]. Only the studies of these seven groups will be examined in detail in this review.

The total synthesis of calyculins has been reviewed by Jacobs and Itching in 1998 [47], and by Pihko and Koskinen in 1999 [48]. The retrosynthetic analyses, as well as the preparation of individual fragments, and the final assembly of the fragments are presented in the following section. In order to compare the different methods, we will first present the preparation of the different fragments separately. Then, the assembly of these fragments to reach the calyculins will be discussed. The total syntheses of calyculins will be presented in chronological order and, for clarity, the different fragments will be described in the same order of publishing year of the total syntheses.

### 5.1. Retrosynthetic analysis

**Scheme 1:**
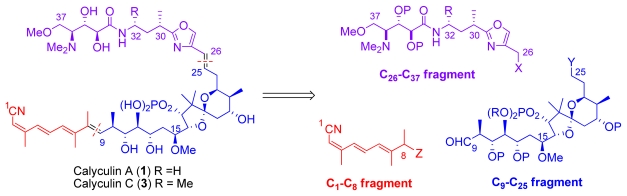
Retrosynthetic analysis of the calyculin skeleton (X, Y and Z denote the functional groups suitable for coupling).

The retrosynthetic analysis of the calyculin skeleton divides it into three fragments: the C_1_–C_8_ tetraene subunit, the C_9_–C_25_ dipropionate spiroketal subunit, and the C_26_–C_37_ amino acid oxazole subunit (Scheme 1).

**Scheme 2:**
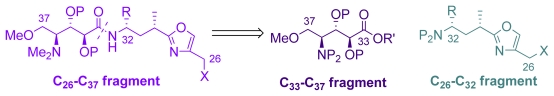
Retrosynthetic analysis of the C_26_–C_37_ fragment (X denotes the functional group suitable for coupling with the fragment C_9_–C_25_).

Further, the C_26_–C_37_ amino acid oxazole subunit is divided into two subunits: the amino acid C_33_–C_37_ subunit and the oxazole C_26_–C_32_ subunit (Scheme 2).

The retrosynthetic analysis and the executions of the different fragments are described in the following sections.

### 5.2. C_1_–C_8_ tetraene fragment

For the synthesis of this fragment, a number of renowned reactions can be highlighted: Horner-Wadsworth- Emmons (HWE), Peterson olefination, Stille coupling, and related Negishi and Suzuki couplings. Evans and co-workers [18] were the first to report that the HWE reaction to couple the entire tetraene is not possible due to the unstable cyano group and tetraenes tendency to isomerisation. The uses of sp^2^-sp^2^ couplings methods such as the Stille coupling were tempting since the double bond geometries of the starting materials are completely retained in the reaction.

**Scheme 3:**
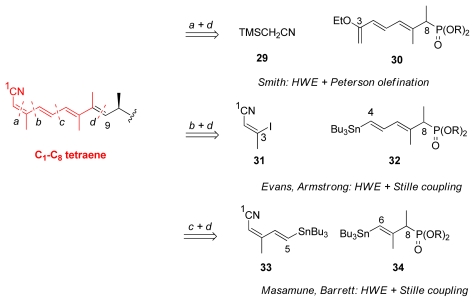
Retrosynthetic analysis of C_1_–C_9_ fragment (X denotes the functional groups suitable for coupling).

Based on the disconnections of the building blocks, the retrosynthetic analysis of tetraene can be divided into three groups (Scheme 3). Smith’s group choose to disconnect in position *a* and *d* which gives nitrile **29** and phosphate **30** [22], whereas Evans’ and Armstrong’s target was to create vinyl iodide **31** and phosphate **32**, *via b* and *d* disconnections [18,21]. Finally, Masamune and Barrett chose *c* and *d* disconnections involving intermediates **33** and **34** [19,23].

In addition, a few other approaches and ideas for the synthesis of this fragment have been published. For example, Negishi *et al.* recently proposed that propyne bromoboration and tandem Pd-catalyzed cross coupling could be used in the synthesis of C_1_–C_8_ fragment [49].

#### 5.2.1. Evans [18]

**Scheme 4:**
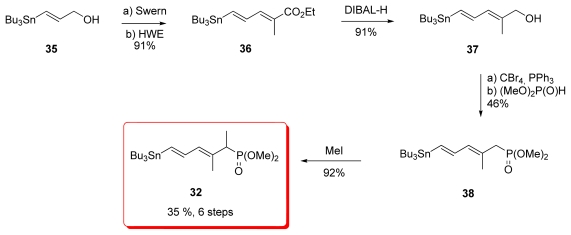
Preparation of phosphonate **32**.

Evans’ synthesis of phosphonate diene **32** began with vinyl stannane **35** which was prepared by reduction of methyl (*E*)-3-(tributylstannyl)-2-propionate (Scheme 4). Swern oxidation followed by HWE reaction gave diene ester **36** as a 19:1 *E:Z* mixture. Reduction of the ester furnished alcohol **37**, which was converted to phosphonate **38** by the Michaelis-Becker method. Final methylation of **38** completed the synthesis of targeted phosphonate **32**.

#### 5.2.2. Masamune [19]

**Scheme 5:**
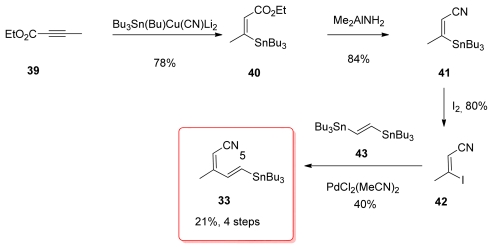
Preparation of stannane **33** by Masamune.

The fact that tributylstannyl moieties can be easily converted to the corresponding iodide was utilized in the synthesis of the tetraene fragment by Masamune *via* fragments **33** and **34**. However, the synthesis of **33** has not been published, and the preparation described here has been found in the PhD thesis of S. A. Filla [50]. Addition of Lipschutz higher cuprate Bu_3_SN(Bu)Cu(CN)Li_2_ to ethyl butynoate **39** occurred regioselectively to furnish the (*Z*)-enoate **40** in good yield (Scheme 5). Treatment of **40** with the Weinreb reagent produced nitrile **41**. Tin-iodide exchange gave vinyl iodide **42** whose Stille coupling with *trans*-1,2-bis(tri-*n*-butylstannyl)ethylene (**43**) produced the expected stannane **33** in a modest 40% yield.

The protected 3-butyl-2-ol **44** was reacted with methylcopper (I) reagent followed by iodination to give vinyl iodide **45**. (Scheme 10) This was converted to phosphonate **46** by an Arbuzov reaction and subsequent tin-iodine exchange with CuSnBu_3_ yielded **34**. This last step appeared to be the weakest link in this part, lowering the global yield.

#### 5.2.3. Shioiri [20]

**Scheme 7:**
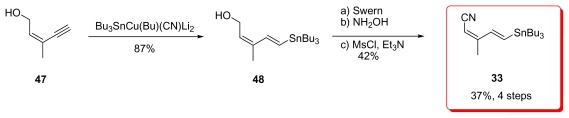
Preparation of stannane **33** by Shioiri.

Shioiri group’s strategy was to introduce the whole tetraene, actually the C_1_–C_12_, as a single moiety to the rest of the target molecule. Stannyl cupration of alcohol **47** was the key step, affording the stannyl derivative **48** in 87% yield. Further conversion of the alcohol to the corresponding nitrile generated **33** (Scheme 7).

**Scheme 8:**
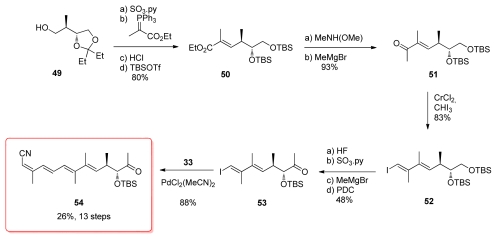
Preparation of tetraene **54.**

The synthesis of the other coupling partner began with alcohol **49** which was readily available from l-(+)-tartrate (Scheme 8). Parikh-Doering oxidation and subsequent HWE reaction were followed by conversion of the acetonide to the bis-TBS ether derivative **50**. Weinreb amide formation and reaction with methyl magnesium bromide furnished methyl ketone **51**, which was converted to (*E*)-vinyl iodine **52** by use of the Takai and Utimoto’s chromium reagent. Selective deprotection of the primary TBS group was followed by conversion of the resulting alcohol to the corresponding methyl ketone **53**. Final Stille coupling of **53** with stannane **33** produced **54**. Shioiri’s group has also published another strategy for the synthesis of this subunit; however, this method has not been used in the formal total synthesis [49].

#### 5.2.4. Smith [22,51]

**Scheme 9:**
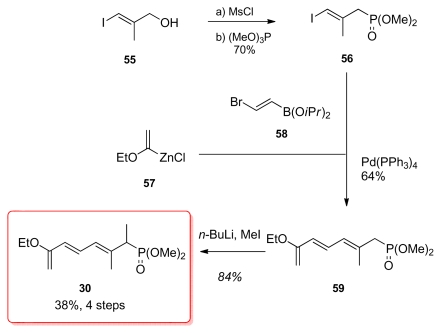
Preparation of phosphonate **30.**

Smith *et al*. began by synthesizing the iodo phosphonate **56** in two steps from allylic alcohol **55** (Scheme 9). This was followed by a two stage one-pot coupling of the three components. The first part, the Negishi coupling of the organozinc compound **57** with bromoboronate **58** was followed by a Suzuki coupling with iodide **55** affording phosphonate **59** in 64% yield. Final methylation of **59** furnished **30**.

#### 5.2.5. Armstrong [21,52]

**Scheme 10:**
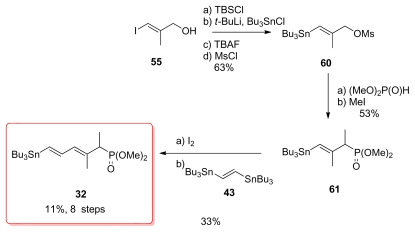
Preparation of phosphonate **32**.

Armstrong’s synthesis of the tetraene fragment also began with the iodo alcohol **55** and proceeded successfully using classical transformations to generate phosphonate **61**, which unfortunately appeared to be too unreactive toward hindered aldehydes (Scheme 10). For this reason, the authors decided to convert **61** to diene **32**. Unfortunately, this transformation proceeded with a low yield of 33% over two steps, lowering the overall yield.

#### 5.2.6. Barrett [23,53]

For the preparation of stannane **33**, Barrett *et al.* used a strategy similar in every aspect to the one presented earlier by Masamune; however, as mentioned before, Masamune’s results were not published. Conjugate addition of tributylstannyl cuprate to ethyl butynoate **39** gave (*Z*)-enoate **40**. Conversion of ethyl ester of **40** to the corresponding nitrile **41** was achieved *via* the amide in 2 steps. Metal-halogen exchange furnished vinyl iodide **42** and Stille coupling with **43** were the lasts steps for the preparation of **33**.

**Scheme 12:**
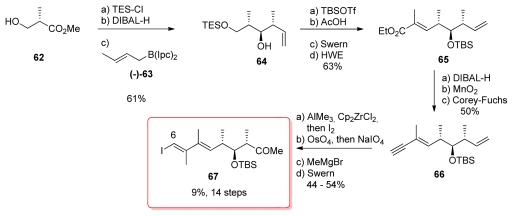
Synthesis of vinyl iodide **67**.

The key idea of the synthesis of the C_6_-C_14_ fragment was to construct the vinyl iodide **67** *via* methyl zirconation–iodonolysis of alkyne **66** using Negishi’s procedure (Scheme 12). The synthesis started with commercially available methyl (*S*)-3-hydroxy-2-methylpropanoate (**62**) which was efficiently converted to homoallylic alcohol **64** by Brown’s homologation with (−)-**63** [54,55], setting the stereochemistry at C_11_ and C_12_ with excellent diastereomeric excess (>96%). Further standard steps led to ester **65**, which was then reduced to aldehyde and homologated to the corresponding alkyne **66** using the Corey-Fuchs protocol. Methylzirconation-iodinolysis of **66** furnishing the corresponding vinyl iodide and final formation of the methyl ketone at C_14_ were the final steps for the preparation of **67**.

#### 5.2.7. Koskinen [40]

**Scheme 13:**
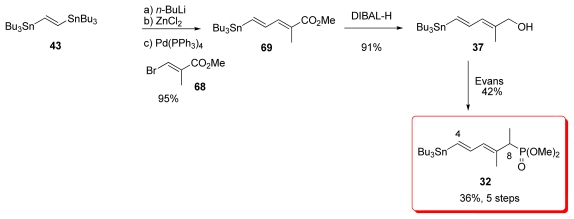
Preparation of **32** by Koskinen.

Koskinen *et al.* reported a short and efficient synthesis of alcohol **37**, which was used by Evans for the preparation of phosphonate **32** (Scheme 13). Sequential treatment of distannyl compound **43** with *n*-BuLi and ZnCl_2_, followed by Pd-catalyzed Negishi coupling with bromo-ester **68**, furnished diene **69** in 95% yield. Reduction with DIBAL-H afforded the alcohol **37**. Introduction of the phosphonate moiety included bromination, Michaelis-Becker reaction and final methylation, following the procedure published by Evans *et al.* [18] gave **32**.

### 5.3. Synthesis of the C_9_–C_25_ dipropionate-spiroketal subunit

The C_9_–C_25_ spiroketal-propionate subunit forms the core of the calyculins. With eleven stereocenters, a phosphate-bearing spiroketal, and *anti, anti, anti* dipropionate segment, the synthesis of this fragment is most challenging.

**Scheme 14:**
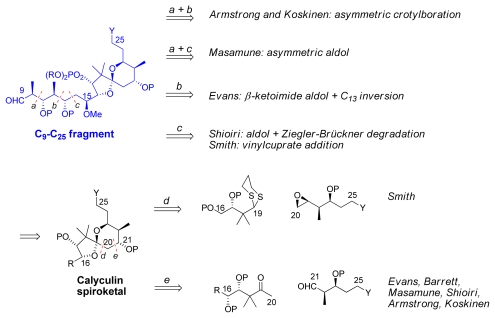
Retrosynthetic analysis of C_9_-C_25_ fragment. (Y denotes the functional groups suitable for coupling and P protective groups).

The construction of the C_9_–C_25_ spiroketal-propionate subunit can be divided into four groups (Scheme 14). Armstrong’s [21] and Koskinen’s group [44–46] chose to introduce the dipropionate in two parts and to use Brown’s asymmetric crotylborane chemistry, while Masamune *et al.* selected the asymmetric aldol strategy [19]. In contrast, Evans *et al*. used a chiral β-ketoamide aldol methodology [18]. Shioiri *et al.* used the Ziegler-Brückner aldol-oxidative degradation method [20] and Smith’s group chose to use vinyl cuprate–epoxide coupling [22].

For the disconnection on the spiroketal, Smith and co-workers strategy was to create the C_19_-C_20_ bond by addition of a dithiane to an epoxide (*via* disconnection *d*). All the other groups decided to use more or less similar aldol-type strategy to create the C_20_–C_21_ bond (disconnection *e*).

Trost and co-workers have also published a synthesis of the C_15_–C_24_ spiroketal core; the synthesis being based on their methodology on ruthenium-catalyzed cyclization and allyl alcohol addition process [56].

#### 5.3.1. Evans [18]

**Scheme 15:**
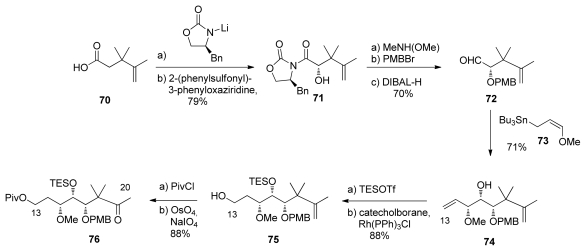
Preparation of ketone **76**.

The authors exploited their studies on the oxazolidone chiral auxiliaries in this synthesis, creating six of the eleven stereocentres with this method. The synthesis of the spiroketal fragment began with the known acid **70**, which could be prepared from diethyl isopropylidenemalonate in three steps (the yields of these early transformations were not reported in the original procedure) [57]. Compound **70** was converted to the (*S*)-phenylalanine-derived oxazolidone, followed by auxiliary-based asymmetric hydroxylation at C_17_ which afforded **71** as a single diastereomer. Removal of the chiral auxiliary and PMB protection at C_17_ were followed by chelation controlled addition of methoxyallylstannane **73** to aldehyde **72**, affording alcohol **74** as a 7.5:1 mixture of diastereomers. Silylation and regioselective Rh-catalysed hydroboration gave alcohol **75**. Finally, pivaloyl protection and oxidative cleavage of the double bond afforded ketone **76** (Scheme 15).

**Scheme 16:**
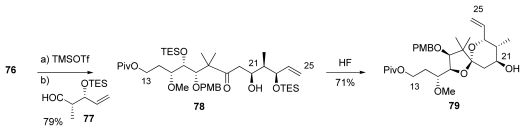
Formation of spiroketal **79**.

Mukayama aldol coupling of between **76** and **77**, prepared in 3 steps *via* classical methods, afforded **78** as a single diastereomer in 80% yield. Spiroketal formation was effected with acid catalysis, furnishing **79** in a 5:1 ratio of diastereomers (Scheme 16).

**Scheme 17:**
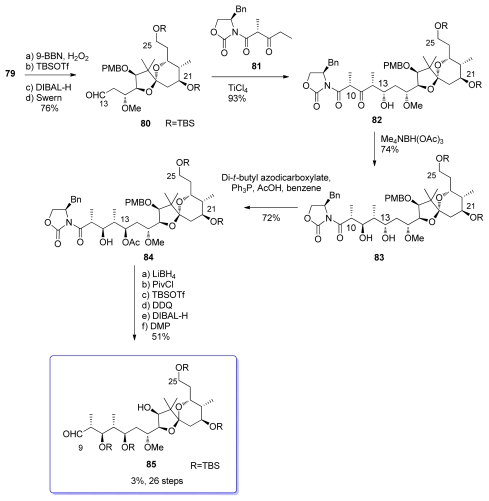
Preparation of **85**.

Spiroketal **79** was then hydroborated and TBS protected at C_25_, followed by pivalate removal and Swern oxidation at C_13_, resulting in the formation of aldehyde **80** (Scheme 17). Addition of **80** to the titanium enolate derived from β-ketoamide **81** provided the *syn, syn* adduct **82** exclusively. A*nti* selective reduction at C_11_ was then performed, yielding diol **83**. The configuration of the C_13_ alcohol had then to be inverted *via* a Mitsunobu reaction to give **84**. Finally, standard transformations provided aldehyde **85**, which represents the C_9_–C_25_ subunit of calyculins.

#### 5.3.2. Masamune [19]

**Scheme 18:**
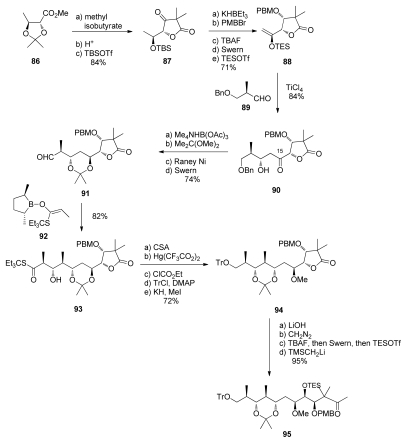
Preparation of **95**.

Masamune *et al.* chose to create the C_10_–C_13_ dipropionate segment before constructing the spiroketal (Scheme 18). The synthesis began with d-threonine derivative **86**. Claisen condensation with methyl isobutyrate, lactonisation, and TBS protection gave **87**. Reduction at C_17_ with KHBEt_3_, with >10:1 diastereselectivity, PMB protection, and conversion to silyl enol ether provided **88**. Then, the titanium-chelated Mukaiyama aldol addition of **88** to aldehyde **89** provided **90** with a 10:1 diastereoselectivity. Reduction at C_15_ with Me_4_NHB(OAc)_3_, diol protection, and a debenzylation-oxidation sequence provided aldehyde **91**.

The authors exploited their studies with chiral borolanyl triflates in asymmetric aldol reactions. The advantage of these reagents is that the intrinsic Felkin bias can be overridden in aldol reactions with chiral aldehydes. This was applied to the construction of the C_10_–C_13_ *anti*, *anti*, *anti* dipropionate. Reacting aldehyde **91** with enolate **92** produced **93** with an excellent 12:1 diastereoselectivity. Acetonide migrations to the more stable *syn* adduct, reduction of the thioester, protection of the alcohol, and C_15_ methylation provided compound **94**. The standard final four steps completed the preparation of the C_9_-C_20_ methyl ketone **95**.

**Scheme 19:**
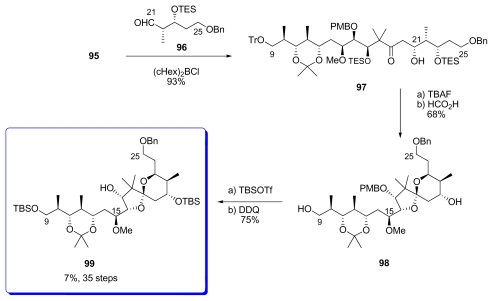
Preparation of spiroketal **99.**

The key aldol reaction between **95** and aldehyde **96** was performed in the presence of bulky (cHex)_2_BCl, leading to the exclusive formation of **97**, without any trace of its C_21_ epimer (Scheme 19). Desilylation and treatment with formic acid provided spiroketal **98** as a single diastereomer. TBS protection and removal of PMB constituted the last steps of the C_9_–C_25_ fragment **99**.

#### 5.3.3. Shioiri [20,58]

Shioiri’s group formal total synthesis of calyculin A consisted of a variety of studies and different strategies. The synthesis of C_14_–C_20_ ketone **105** began from diethyl l-tartrate **100** which was converted in five steps to the key intermediate **101.** Aldol reaction with ketene acetal **102**, in the presence of chiral borane reagent **103**, stereoselectively produced **104**, which was then easily converted to the corresponding methyl ketone **105**.

**Scheme 21:**
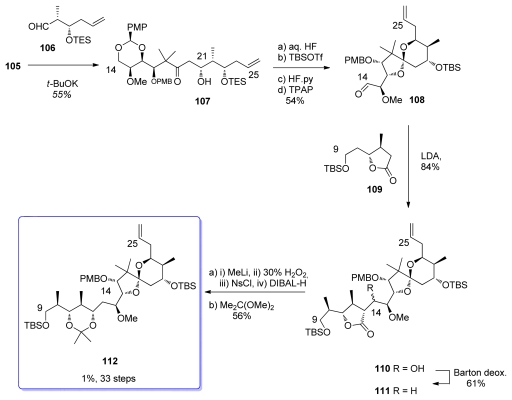
Synthesis of spiroketal **112**.

The aldol reaction between **105** and aldehyde **106**, easily prepared from dimethyl l-malate, proceeded with an excellent diastereoselectivity to give **107** in a 18:1 ratio (Scheme 21). Interestingly, only the potassium enolate of **105** gave satisfactory results in the formation of the *syn* aldol adduct, the lithium and sodium enolates giving only poor diastereoselectivity. Spiroketalization was then performed in aqueous HF. Further protection-deprotection sequence followed by TPAP oxidation gave aldehyde **108**. Coupling of **108** with the enolate of C_9_–C_13_ lactone **109** furnished **110** as a mixture of diastereomers. Barton deoxygenation of the hydroxyl group at C_14_ furnished **111**, together with its C_13_ epimer in a 4:1 ratio (the latter being further epimerized with MeLi to give the desired **111**). Degradation of the γ–lactone to the 1,3-acetonide was performed using Ziegler-Brückner conditions and further protection of the diol to gave **112**.

#### 5.3.4. Smith [22,59,60]

**Scheme 22:**
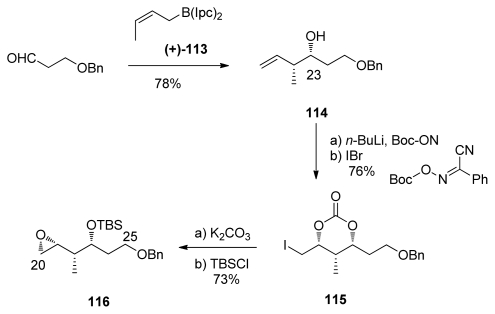
Synthesis of epoxides **116**.

Smith published the synthesis of the spiroketal core of *ent*-calyculin A in 1991, using a novel dithiane-epoxide coupling strategy. Brown’s crotylation on 3-benzyloxypropanal, using (*Z*)-crotylboron reagent (+)-**113**, furnished alcohol **114** in 99% enantiopurity (Scheme 22). Boc-protection and electrophilic cyclization in the presence of IBr afforded the *syn, syn* carbonate **115**, with good α/β selectivity of 13.9/1. The synthesis of C_20_–C_25_ coupling moiety was finished with cleavage of the carbonate group, and TBS protection to give epoxide **116**.

**Scheme 23:**
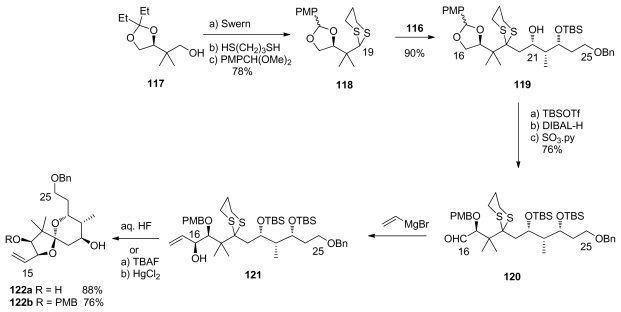
Preparation of spiroketal **122**.

The C_16_-C_19_ dithiane moiety **118** was easily prepared from alcohol **117**, *via* consecutive Swern oxidation, dithiane formation and diol protection (Scheme 23). Key coupling was performed by metalation of **118** with *n-*BuLi followed by addition of DMPU and epoxide **116**, to furnish alcohol **119**. Silyl protection, acetal reduction, and Parikh-Doering oxidation then afforded aldehyde **120**. The stereochemistry of C_16_ was created by chelation-controlled addition of vinylmagnesium bromide to **120** to give alcohol **121** with a >20:1 diastereoselectivity. Two different conditions for the spirocyclisation were employed. The first, using aqueous HF, produced **122a** as a single diastereomer in 88% yield but resulted in the loss of PMB group at C_17_. To retain this useful protecting group, an alternative sequence was applied. Sequential treatment of **121** with TBAF and HgCl_2_/CaCO_3_ afforded a mixture of **122b** and its C_19_ epimer. Fortunately, the latter could be quantitatively converted to **122b** upon exposure to *p*-TsOH, with a global yield of 76%.

After extensive experimentation, the authors found out that Payne epoxidation of **122b** occurred in good *syn* diastereoselectivity and yield (9.5:1, 89%) to give **123**. After TBS-protection, the epoxide was coupled with the vinyl cuprate derived from **124** giving access to **125** as a single diastereomer in 83% yield. Methylation of the C_15_ hydroxyl group of **125**, and oxidation of the alkene were followed by DIBAL-H reduction to provide **126** with >12:1 diastereoselectivity at C_13_ (probably *via* internal hydride delivery by prior coordination at the C_15_ methoxy group). Finally, protecting group manipulations provided compound **127**.

#### 5.3.5. Armstrong [21,52]

**Scheme 25:**
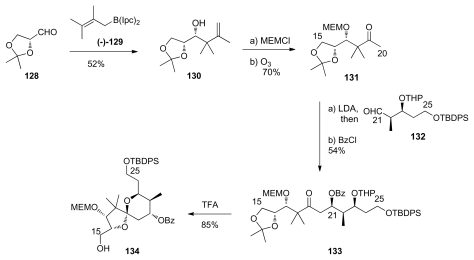
Preparation of spiroketal **134.**

Armstrong’s group used Brown’s chiral allylborane reagents in their synthesis of C_9_–C_25_ fragment. The synthesis of spiroketal core starts with the reaction of d-glyceraldehyde **128** with the enantiopure allylboron reagent (−)-**129** (Scheme 25). This addition occurred in good yield and excellent stereoselectivity to provide **130**. After MEM-protection and ozonolysis, ketone **131** was obtained. Aldol reaction between **131** and aldehyde **132** and further benzoyl protection furnished compound **133**. Upon exposure to TFA, **133** underwent the expected tandem deprotection-spirocyclisation to give the desired spiroketal **134** as a single enantiomer.

**Scheme 26:**
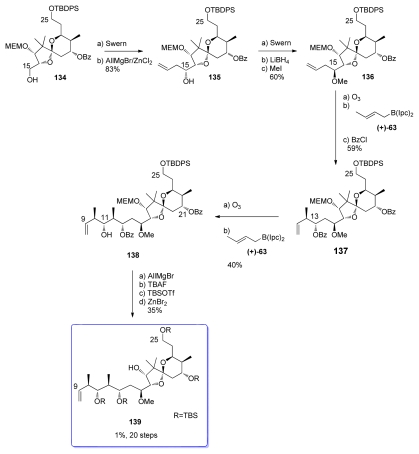
Preparation of **139**.

Swern oxidation of alcohol **134** was followed by reaction with allylmagnesium bromide in the presence of ZnCl_2_ to give homoallylic alcohol **135** (Scheme 26). Unfortunately, the Felkin-Ahn mode was followed for this addition, resulting in the formation of the stereochemistry at C_15_ opposite to that present in the natural product. Inversion at C_15_ was performed by consecutive oxidation-reduction. Finally, the new C_15_ hydroxyl was methylated to give **136**. Ozonolysis of **136** afforded the corresponding aldehyde that, upon submission to an asymmetric Brown’s crotylboration with (+)-**63** and benzoyl protection, gave olefin **137** as a single diastereomer. Second ozonolysis gave the corresponding aldehyde which was the substrate for a further Brown’s crotylation. Unfortunately, this step proceeded with very low diastereoselectivity; the expected *anti, anti, anti* adduct **138** being isolated in only 40% yield; the *anti, syn, anti* isomer being isolated in 30%. This disappointing result led the authors to study the influence of the different protecting groups at C_25_, C_21_ and C_13_ on the crotylation process. Unfortunately, all the other protecting group combination gave lower yield of the expected **138**, the undesired isomer always being the main product. The tetra-TBS protected compound **139** was then prepared by simple protection group conversion of **138**, followed by MEM-deprotection.

#### 5.3.6. Barrett [23,61]

**Scheme 27:**
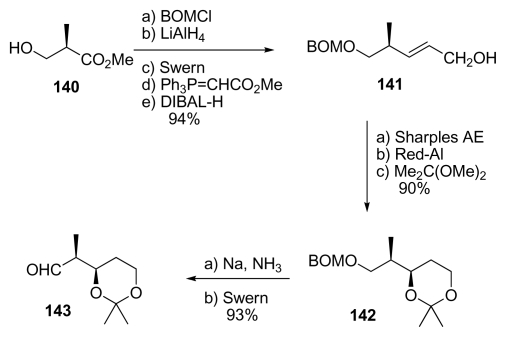
Preparation of aldehyde **143**.

Barrett *et al.* also used the Brown’s crotyl boration reagents for the synthesis of the spiroketal moiety of calyculins. The synthesis began with ester **140** which was converted to allylic alcohol **141** (Scheme 27). Sharpless asymmetric epoxidation, ring opening and protection of the resulting diol furnished acetonide **142**. Deprotection of the benzyloxymethyl ether, and subsequent Swern oxidation completed the synthesis of aldehyde **143**.

**Scheme 28:**
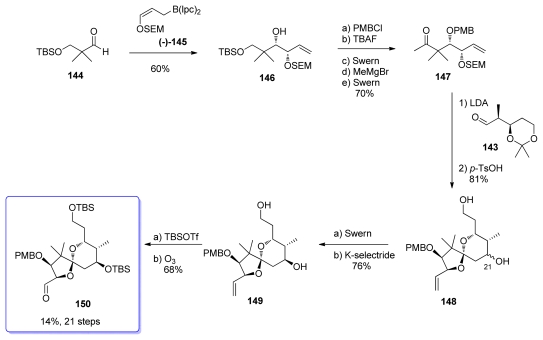
Preparation of spiroketal **150**.

Addition of (*Z*)-borane (−)**-145** to aldehyde **144** proceeded effectively to yield the *syn* adduct **146** with >95% stereocontrol (Scheme 28). Transformation of **146** to the corresponding methyl ketone **147** was then carried out using standard transformations. Key aldol reaction between **147** and aldehyde **143** yielded, after acidic treatment, to the spiroketal **148**, as a 2:1 mixture of C_21_ epimers. Swern oxidation followed by K-selectride reduction furnished diol **149** as a single diastereomer. Final protection-oxidation steps led to compound **150**.

#### 5.3.7. Koskinen [42–46]

**Scheme 29:**
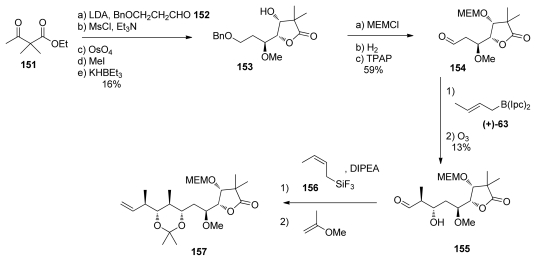
Preparation of **157.**

The Koskinen group has presented separate studies for the preparation of the dipropionate and the spiroketal moieties. The key lactone **153** was prepared in five steps from ketoester **151** and aldehyde **152** (Scheme 29). MEM-protection, benzyl deprotection and further oxidation provided aldehyde **154**. This aldehyde was subjected to a Brown’s crotylation, affording the expected homoallylic alcohol in a 6:1 separable mixture of diastereomers, in favor of the expected product. Further ozonolysis furnished aldehyde **155**, which was, in turn, subjected to a crotylation reaction. Based on previous studies by Armstrong [21,52] showing that a second Brown’s crotylation on similar substrates gave only poor selectivity, the authors decided to use the Roush (*Z*)-crotyl trifluorosilane **156** for this reaction [62]. This transformation pleasingly afforded a single isomer and further acetonide protection furnished **157**, whose analysis proved the *anti, anti, anti* relationships in the stereotetrad. This strategy proved to be efficient in terms of selectivity, but suffers from poor yields. Current studies in our lab recently showed that this methodology could be improved and much better yields were obtained on new substrates used in the course of the synthesis of calyculins (unpublished results).

**Scheme 30:**
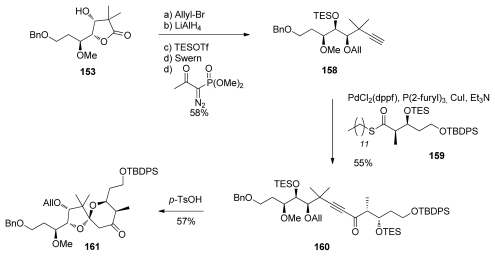
Preparation of spiroketal **161.**

Koskinen also recently published a preparation of the C_13_–C_25_ spiroketal core of calyculins (Scheme 30) [46]. Lactone **153** was allyl-protected, reduced with LiAlH_4_, and the diol protected as a TES ether. Further selective deprotection-oxidation of the primary alcohol, and homologation of the aldehyde to the corresponding alkyne using the Ohira-Bestmann protocol produced **158** in good yield. Key coupling between acetylene **158** and thioester **159** furnished ynone **160**. This was subjected to acidic treatment, leading to TES-deprotection followed by a double intramolecular hetero-Michael addition (DIHMA) to yield **161** as a single enantiomer. The DIHMA spiroketalisation differs from all the other methods described for this fragment.

### 5.4. Syntheses of the C_26_-C_32_ oxazole fragment

Compared to the synthesis of C_9_-C_25_ spiroketal-propionate subunit, the C_26_–C_32_ oxazole fragment seems to be less difficult; however, formation of the C_30_ stereocenter creates challenges. Construction of the suitably substituted oxazole in good yield without epimerization proved challenging.

**Scheme 31:**
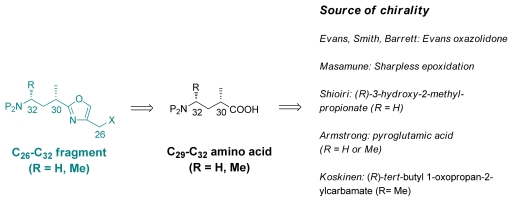
Retrosynthetic analysis of C_26_–C_32_ fragment (X denotes the functional groups suitable for coupling and P the protective group).

Retrosynthetically, the C_26_-C_32_ can be simplified to a chiral aminoacid (Scheme 31). The Evans’ oxazolidone was used as source of chirality by Evans [18], Smith [22], and Barrett [23] whereas Masamune used the Sharpless epoxidation [19]. Shioiri decided to use a hydroxy acid as the starting material [20]. Finally, Armstrong started from l-pyroglutamic acid [21], while Koskinen used *d*-alaninal [39].

#### 5.4.1. Evans [18]

**Scheme 32:**
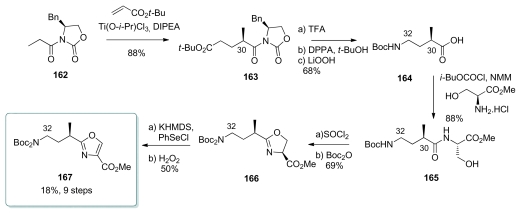
Preparation of oxazole **167**.

The key step of the oxazole synthesis was the diastereoselective Michael addition of *N*-propionyloxazolidone **162** to *tert*-butyl acrylate, setting up the correct stereochemistry for **163** at C_30_ in 88% yield with >95:5 diastereoselectivity (Scheme 32). Cleavage of the *tert*-butyl ester followed by Curtius rearrangement afforded amino acid **164**, which was coupled with l-serine methyl ester to give **165**. Cyclisation with thionyl chloride afforded the corresponding oxazoline **166**. Finally, oxidation by trapping the enolate of **166** with PhSeCl followed by oxidative elimination afforded oxazole **167**.

#### 5.4.2. Masamune [19,63]

**Scheme 33:**
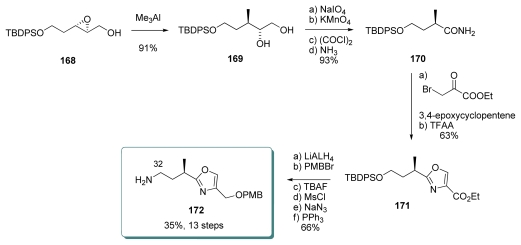
Preparation of oxazole **172**.

The starting point for the oxazole fragment **172** was the epoxy alcohol **168**, obtained by Sharpless asymmetric epoxidation of the corresponding allylic alcohol; unfortunately, no details about the yield or selectivity were reported (Scheme 33). Regioselective ring-opening of **168** with Me_3_Al provided the diol **169**, with the requisite stereochemistry at C_30_. Oxidative cleavage of **169**, further oxidation to acid and conversion to amide furnished **170**. Treatment of **170** with ethyl bromopyruvate furnished, after dehydration, oxazole **171**. To avoid epimerization, this reaction had to be carried out in the presence of 3, 4-epoxycyclopentene. Final preparation of oxazole **172** was achieved with standard transformations.

#### 5.4.3. Shioiri [20,64]

**Scheme 34:**
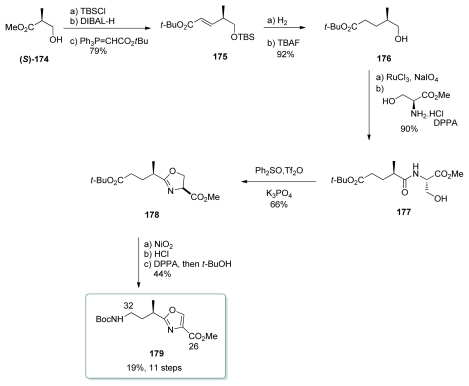
Preparation of oxazole **179.**

For the synthesis of oxazole fragment of calyculin A, Shioiri *et al.* used methyl (*S*)-3-hydroxy-2-methylpropionate [(*S*)-**174**]. TBS protection, DIBAL-H reduction to the corresponding aldehyde, and Wittig olefination gave the unsaturated ester **175** (Scheme 34). This was followed by hydrogenation of the double bond, and cleavage of the TBS-deprotection to yield alcohol **176**. Oxidation of **176** to the corresponding acid and coupling with l-serine methyl ester gave **177**. For the conversion to the oxazoline **178**, the authors applied their own method which uses triflic anhydride, diphenyl sulfoxide and potassium phosphate. Oxazoline **178** was obtained in 66% yield, without any epimerization in the process. Oxidation with NiO_2_ provided the corresponding oxazole. Finally, after removal of the *tert*-butyl ester, Curtius rearrangement gave the amino ester **179**. This synthesis afforded the enantiomer of the fragment present in the natural product. However, the enantiomer of **179** can easily be obtained by using (*R*)-3-hydroxy-2-methylpropionate [(*R*)-**174**] as starting material.

The authors presented also a method for the preparation of the C_26_–C_32_ oxazole part of calyculin C, which adds an extra methyl group at C_32_ [57]. This method relied on the asymmetric ring opening of a prochiral cyclic anhydride. However, this strategy has not been used in any total synthesis and therefore will not be described here in detail.

#### 5.4.4. Smith [22,51]

**Scheme 35:**
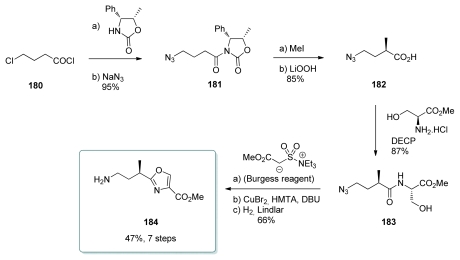
Preparation of oxazole **184.**

The synthesis started with 4-chlorobutyl chloride (**180**) which was converted to oxazolidinone **181** (Scheme 35). Methylation (87% yield, 95% diastereoselectivity) followed by removal of the chiral auxiliary furnished acid **182**. Condensation with l-serine methyl ester in the presence of diethylcyanophosphonate (DECP) as the coupling agent yielded amide **183**. Exposure of **183** to Burgess reagent followed by Narrish-Singh oxidation produced the desired oxazole ring. Finally, azide reduction furnished **184** in good yield and without epimerisation.

#### 5.4.5. Armstrong [21,65]

**Scheme 36:**
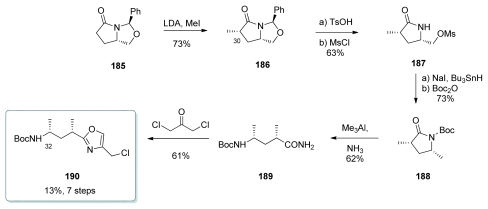
Preparation of oxazole **190.**

Comparing to other syntheses of this subunit, Armstrong targeted calyculin C, which contains one additional methyl at C_32_. Bicyclic *N,O*-acetal **185**, which was prepared from (*S*)-pyroglutamic acid, was methylated at C_30_ to give **186** in a 80:20 ratio of diastereomers (Scheme 36). Acetal hydrolysis followed by mesylation afforded lactam **187**. Radical deoxygenation of the *in situ* formed iodide and Boc-protection yielded **188**. Ring opening by Me_3_Al in the presence of ammonia furnished the open-chain amide **189**. Finally, oxazole **190** was obtained by reaction of **189** with 1,3-dichloroacetone.

#### 5.4.6. Barrett [23,66]

**Scheme 37:**
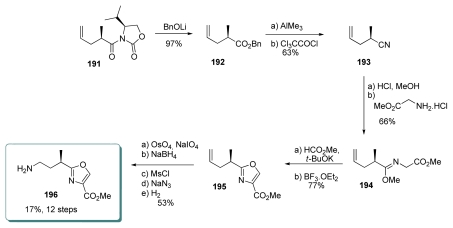
Preparation of oxazole **196**.

Barrett *et al*. synthesized the oxazole unit by using a modified Cornforth-Meyers approach (Scheme 37). The synthesis started with oxazolidinone **191**, which was reacted with lithium benzyloxide to give ester **192**.

Nitrile **193** was obtained by submitting **192** to Me_3_Al and dehydration. Addition of MeOH and HCl to **193** produced an intermediate imidate ion, which reacted with glycine methyl ester to produce **194**. The Cornforth-Meyers procedure, using methyl formate in the presence of *t*-BuOK and BF_3_.OEt_2_, provided oxazole **195**. Final stages transformed the terminal alkene of **195** to the corresponding primary amine **196** in 5 steps. No racemization was observed during the entire process.

#### 5.4.7. Koskinen [39]

**Scheme 38:**
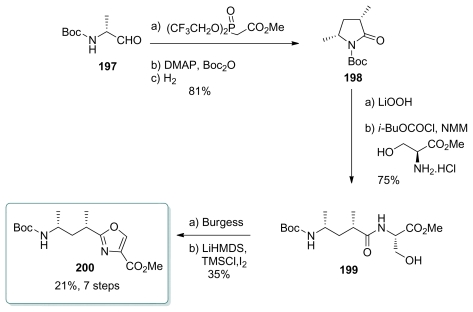
Preparation of oxazole **200.**

Koskinen group’s strategy towards calyculin C was to use cyclic stereocontrol to create the *syn*-isomer of C_26_–C_32_ (Scheme 38). *d*-Alaninal derivative **197** was subjected to Still-Gennari modification of HWE olefination to give the corresponding (*Z*)-enoate, whose cyclization under Ragnarsson-Grehn conditions (Boc_2_O, cat. DMAP) and hydrogenation furnished lactam **198**, together with 9% of its *anti* diastereomer. Hydrolysis of **198** followed by coupling with l-serine methyl ester afforded amides **199** (at this stage, the *anti* diastereomer could be separated by chromatography). Conversion to oxazole **200** was achieved by treatment with Burgess reagent to give the oxazoline, followed by oxidation. This oxidation proved to be difficult, and the best results were obtained by using either CuBr_2_/HMTA/DBU as discussed earlier [22,51] or by temporary TMS protection of the carbamate hydrogen, deprotonation of the oxazoline and oxidation of the enolate with I_2_. Those two strategies yielded 42% of oxazole **200**.

### 5.5. Syntheses of the C_33_–C_37_ amino acid fragment

**Scheme 39:**
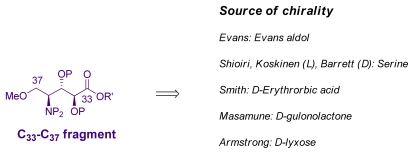
Retrosynthetic analysis of C_33_–C_37_ fragment.

Because of the three chiral centres and possibly reactive amine, the synthesis of C_33_–C_37_ is also a challenging target. For the synthesis of this fragment, most groups used carbohydrates. It can be also noticed that the left half of the fragment resembles serine. This was exploited by the Shioiri, Barrett, and Koskinen groups (Scheme 39) [20,38,65,66].

#### 5.5.1. Evans [18]

**Scheme 40:**
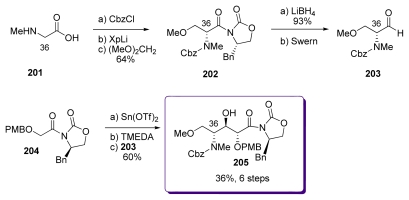
Preparation of **205**.

Sarcosine derived oxazolidinone was prepared from **201** and further alkylated with dimethoxyethane in good yield (80%) and diastereoselectivity (98:2) to produce **202** (Scheme 40). Displacement of the chiral auxiliary with LiBH_4_ followed by Swern oxidation, where Hunig’s base was employed to prevent racemisation, cleanly furnished aldehyde **203**. Enolization of **204** in the presence of Sn(OTf)_2_ and TMEDA followed by addition of aldehyde **203** afforded the expected *anti* aldol **205** in 60% yield. Unfortunately significant amounts of other diastereomers were also formed in the reaction.

#### 5.5.2. Masamune [19,63]

**Scheme 41:**
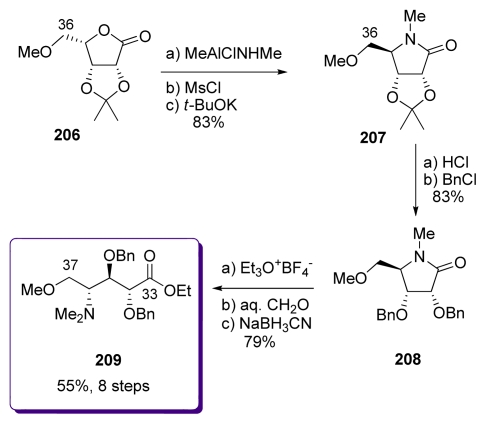
Preparation of **209**.

The synthesis of the aminoacid derivative **209** began with the reaction of lactone **206**, easily prepared from gulonolactone, with the Weinreb’s reagent of methylamine (Scheme 41). The crude hydroxyamine obtained was then mesylated and treated with *t-*BuOK to provide lactam **207**, with reverse stereochemistry at C_35_ to that present in **206**. Cleavage of acetonide liberated the corresponding diol which was protected as the dibenzyl ether **208**. Treatment of **208** with Meerwein’s reagent provided the corresponding imidate, which after hydrolysis, reaction with formaldehyde and reduction with cyanoborohydride, furnished aminoester **209**, the enantiomer of the C_33_–C_37_ fragment present in calyculins. However, in the course of the total synthesis of natural calyculin A, the authors briefly state that the synthesis of the fragment with the correct stereochemistry had been performed with significant improvements compared to **209**.

#### 5.5.3. Shioiri [20,67]

**Scheme 42:**
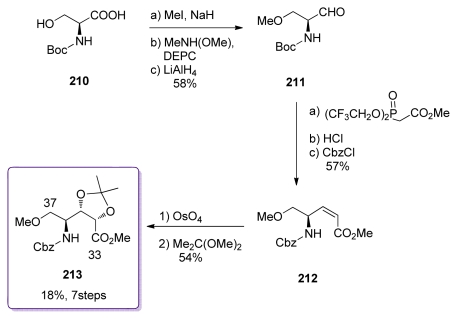
Preparation of ester **213**.

Shioiri *et al.* also published a synthesis of the C_33_–C_37_ fragment. Their strategy was based on OsO_4_ dihydroxylation of the l-serine derived *Z*-alkene **212** (Scheme 42). Boc-l-serine **210** was *O-*methylated and converted to aldehyde **211**. Reaction with Still-Gennari’s phosphonate and Boc replacement by Cbz group furnished the *Z*-enoate **212**. Dihydroxylation in the presence of dihydroquinine *p*-chlorobenzoate produced the corresponding diol (80% total yield, 80:20 diastereoselectivity), which was then protected as its acetonide **213**.

Shioiri’s group has also earlier published a synthesis of enantiomer of the C_33_–C_37_ fragment. Although it was not utilized in the total synthesis, it had a great effect in determining the absolute stereochemistry of the calyculins.

#### 5.5.4. Smith [22,51]

**Scheme 43:**
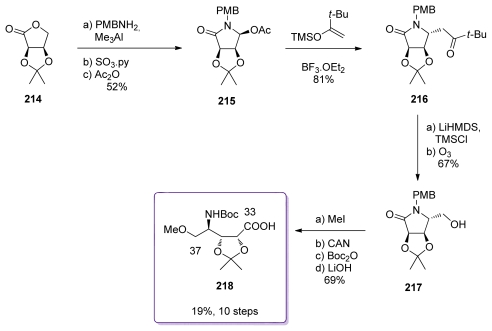
Preparation of **218.**

The starting point of the preparation of the amino acid subunit was commercially available *iso*-propylidene *d*-erythronolactone (**214**, Scheme 43). Treatment of **214** with PMBNH_2_ in the presence of Me_3_Al gave the ring opened hydroxyl amide. Parikh-Doering oxidation of the primary alcohol and dehydration led to the formation of **215**, in a 7:1 anomeric mixture in favor of the β–anomer. In the presence of the TMS-enol ether of pinacolone and BF_3_.OEt_2_, **215** elegantly led to the formation of ketone **216** in 81% yield and as a single diastereomer. Formation of the TMS-silyl ether of **216** followed by reduction provided alcohol **217**. Methylation of the free hydroxyl group of **217**, PMB-Boc protecting group exchange and basic hydrolysis were the last steps for the preparation of **218**.

#### 5.5.5. Armstrong [21,65]

**Scheme 44:**
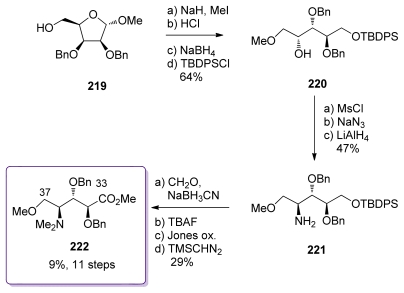
Preparation of **222**.

The synthesis started from alcohol **219**, available in four steps from *d*-lyxose (Scheme 44). *O-*Methylation, acidic hydrolysis followed by mild reduction yielded the corresponding diol which was selectively silyl-protected to give **220**. The C_36_ stereochemistry of **220** is opposite to that found in the natural product. Inversion was achieved *via* mesylation, azidation and reduction to furnish amine **221**. Classical steps of amine-methylation, silyl deprotection and oxidation of the primary alcohol to the corresponding methyl ester completed the preparation of **222**. The overall yield for this fragment remains low mainly because of the difficulties encountered in the Jones oxidation.

#### 5.5.6. Barrett [23,66]

**Scheme 45:**
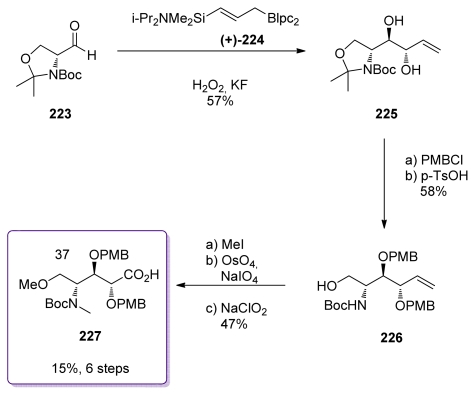
Preparation of **227**.

Like the other fragments in the Barrett’s total synthesis of *ent*-calyculin A, the aminoacid part was also prepared using an allylboration strategy (Scheme 45) [54,55]. Key reaction of Garner’s aldehyde **223** with the silylated allylborane derivative **224** followed by oxidative cleavage of the C-Si bond produced stereospecifically diol **225**, which was then protected as a di-PMB ether. Selective hydrolysis of the isopropylidene ketal gave alcohol **226**. *O-* and *N-*methylation preceeded the oxidative cleavage of the double bond, which was further oxidized to acid to furnish amino acid **227**.

#### 5.5.7. Koskinen [38]

**Scheme 46:**
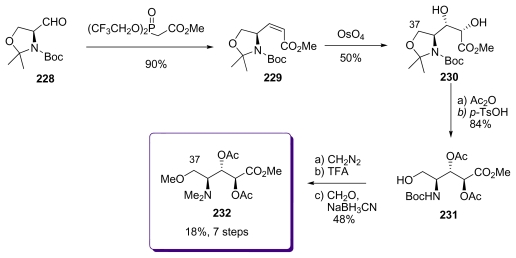
Preparation of **232**.

The synthesis of the aminoacid fragment C_33_-C_38_ by Koskinen *et al.* began with the l-serine derived aldehyde **228** which gave, after treatment with the Still-Gennari phosphonate, the *Z*-enoate **229** (Scheme 46). The key-step for this sequence was the stereospecific dihydroxylation. Taking advantage of the allylic strain of *Z*-olefins enhanced by the presence of the cyclic protecting group pattern, treatment of **229** with OsO_4_ led to the formation of a single diol **230**, whose analysis proved >99% optical purity. After acetate protection, the acetonide was cleaved to amino alcohol **231**. *O-*Methylation, Boc-deprotection and *N*-dimethylation finished this synthesis to give **232**.

### 5.6. Finishing the total synthesis: introduction of phosphonate and assembly of fragments

The syntheses of the four fragments of calyculins have without a doubt created a great challenge to all chemists involved in the synthesis work. However, before the final assembly of the fragments, representing the final judgement of the efficiency of the total synthesis, there was still one problem to solve: the introduction of the C_17_ phosphate group. The C_17_ is placed in very shielded and hindered position in the spiroketal core. It therefore requires a reactive electrophile for the activation, but at the same time it has to be mild enough not to react with other parts of the molecule. It is interesting that even if phosphate groups are common in natural products, techniques for their introduction are still limited.

Because the Evans and Masamune groups were the first ones involved in the total synthesis of calyculins, they performed the pioneering work in that field, by studying the different possibilities protecting phosphate groups [18,19,68]. They both ended up using phosphorous (III) compounds as reactive electrophiles; this technique being used later by all the other groups.

The efforts towards the total synthesis of any natural product are truly tested in the coupling of fragments. Without a good method for that, even the greatest synthesis does not complete its final goal. The completion of the six published total synthesis of calyculins are discussed next.

#### 5.6.1. Total synthesis of *ent*-calyculin A by Evans [18]

The total synthesis published by Evans and co-workers was the first completed synthesis of a member of the calyculin family; however, the molecule obtained appeared to be the enantiomer of calyculin A. Thanks to this work, the absolute configuration of calyculins was finally determined.

**Scheme 47:**
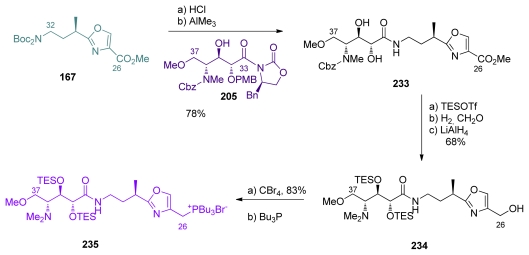
Preparation of the C_26_–C_37_ fragment **235**.

**Scheme 48:**
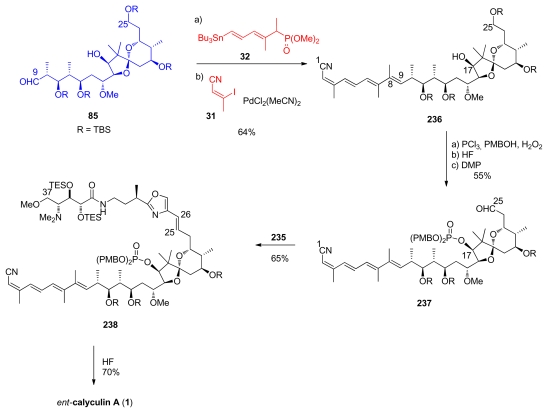
Final steps for the synthesis of *ent*-calyculin A.

The assembly of the fragments is shown in Scheme 47 and Scheme 48. It should be noted that the protection of hydroxyl groups was planned as a *cumulative silicon strategy*. If a protection needed to persist until the end of the synthesis, silicon-based protecting groups were used while more temporary protecting groups were non-silicon based.

To produce the C_26_–C_37_ subunit, the oxazole **167** was first Boc-deprotected and coupled with oxazolidone **205** in the presence of Me_3_Al, pleasingly leading to the formation of the amide bond and PMB-deprotection in the same operation (Scheme 47). Diol **233** was thus obtained and was further TES-protected, which was followed by CBz-removal, reductive methylation of the amine, and reduction of the ester group to furnish **234**. Conversion of the alcohol to the corresponding tributylphosphonium salt, precursor for the Wittig reaction, terminated the preparation of **235**.

The preparation of the C_1_–C_25_ fragment was finished with the HWE reaction between aldehyde **85** and stannyl phosphonate **32** which afforded the corresponding stannyl triene as a 7:1 *E:Z* mixture (Scheme 48). Stille coupling of the triene with vinyl iodide **31** gave the tetraene **236**. The phosphonate at C_17_ was then introduced, by treating **236** with PCl_3_, followed by PMBOH and *in situ* oxidation of the intermediate phosphite with H_2_O_2_. Selective removal of the primary TBS at C_25_ and subsequent oxidation produced aldehyde **237**. Wittig reaction between **237** and the phosphonium salt **235** afforded the protected product **238**. Treatment of **238** by HF finished the first total synthesis of *ent*-calyculin A.

#### 5.6.2. Total synthesis of calyculin A by Masamune [19,63]

**Scheme 49:**
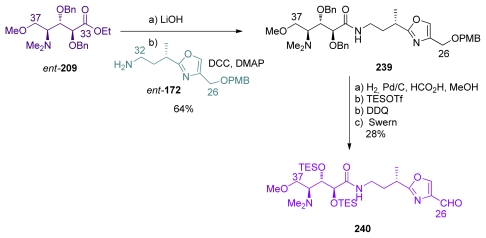
Preparation of C_26_–C_37_ fragment **240**.

Masamune *et al*. published in 1994 the first total synthesis of naturally occurring calyculin A. The assembly of C_26_–C_37_ fragment is described in Scheme 49. Ester *ent*-**209** was saponified and then coupled with amine *ent*-**172** which gave amide **239**. Selective deprotection of the C_34_ and C_35_ benzyl moieties in the presence of C_26_ PMB group was achieved by performing the hydrogenation in a HCO_2_H/MeOH solvent mixture. Further TES-protection of the resulting diol, PMB removal and oxidation to aldehyde produced **240**.

**Scheme 50:**
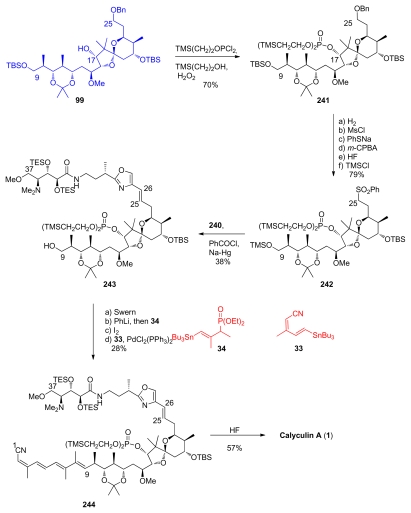
Final steps to calyculin A.

Then, the phosphate introduction at the C_17_ alcohol of spiroketal **99** was carried out (Scheme 50). The authors chose trimethylsilylethyl phosphate ester, a protecting group they had previously studied [68], which was introduced by successively treating **99** with TMSCH_2_CH_2_OPCl_2_, TMSCH_2_CH_2_OH and H_2_O_2_, to yield **241**. To create the C_25_–C_26_ double bond, the Masamune group decided to use Julia-Lythgoe conditions. In this purpose, the benzyl group at C_25_ was removed and conversion of the alcohol to the corresponding sulfone was carried out. Additional protecting group exchange at C_9_ was also performed to give sulfone **242**. Key Julia-Lythgoe olefination between **242** and **240** was then performed, providing **243**. The geometry of the newly formed C_25_-C_26_ double bond was described as being predominantly *E*, however no *E:Z* ratio was reported. Oxidation of the primary alcohol of **243** was followed by reaction with phosphonate **34** and tin/iodide exchange. Stille coupling with stannyl **33** furnished compound **244**. Final HF treatment completed the first total synthesis of natural calyculin A.

#### 5.6.3. Formal total synthesis of calyculin A by Shioiri [20]

The formal total synthesis of *ent-*calyculin A by Shioiri and co-workers converges on the C_9_–C_37_ intermediate **243** in the Masamune’s synthesis. This synthesis allows a comparable overall yield with considerably shorter synthesis.

**Scheme 51:**
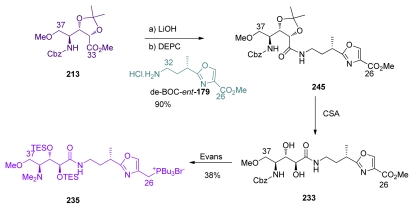
Preparation of C_26_–C_37_ **235** by Shioiri.

The C_26_–C_37_ fragment was synthesized efficiently from **213** and de-BOC-*ent-***179** (Scheme 51). The coupling of the fragments C_26_–C_32_ and C_33_–C_37_ in the presence of DEPC furnished amide **245** in 90% yield. Acetonide deprotection furnished diol **233**, which was converted to phosphonium **235** following Evans procedure [18].

**Scheme 52:**
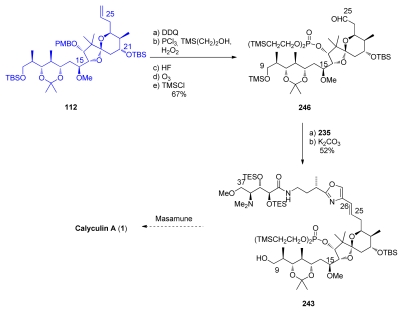
Preparation of **243**.

PMB-removal at C_17_ on **112** was followed by phosphorylation, C_9_ protecting group exchange and ozonolysis at C_25_ to give aldehyde **246** (Scheme 52). Wittig reaction between **246** and phosphonium salt **235**, followed by TMS-deprotection at C_9_ furnished compound **243**, the intermediate previously reported by Masamune.

#### 5.6.4. The total synthesis of *ent*-calyculin A and B by Smith [22,51,69]

**Scheme 53:**
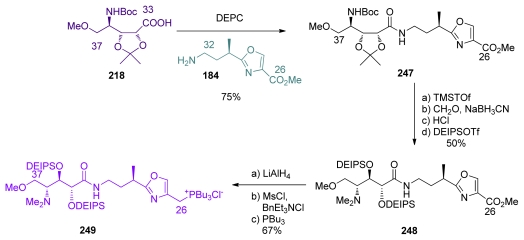
Preparation of C_26_–C_37_ fragment **249.**

Smith and co-workers tenacious work afforded the total syntheses of *ent*-calyculin A and B in 1999. The coupling of C_33_–C_37_ oxazole **184** and C_26_–C_32_ acid **218** was carried out in the presence of DEPC, as described by Shioiri, to give **247** (Scheme 53). Boc-deprotection was followed by methylation of the amine, and acetonide deprotection liberated diol which was protected with DEIPS group, to yield **248**. Finally, conversion of the methyl ester at C_26_ to the phosphonium salt **249** was carried out *via* the corresponding chloride.

**Scheme 54:**
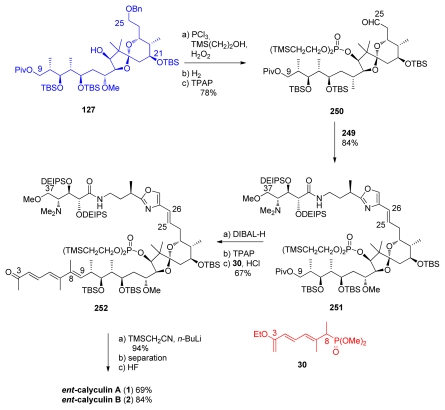
Final steps to *ent-*calyculin A and B.

Phosphate introduction at C_17_ of **127** was followed by benzyl deprotection and oxidation to furnish aldehyde **250** (Scheme 54). Wittig olefination between **250** and phosphonium chloride **249** provided **251** as a 9:1 *E:Z* mixture in 84% yield. Pivaloyl-removal at C_9_ and subsequent oxidation of **251** furnished the corresponding aldehyde, which was olefinated *via* HWE reaction with phosphonate **30** to give methyl ketone **252**, after acidic treatment, in excellent 92% yield and 15:1 *E:Z* ratio. Peterson olefination of **252** furnished a 1.7:1 mixture of the *E* and *Z* isomers, which respectively corresponds to protected *ent*-calyculin A and *ent*-calyculin B. After chromatographic separation, final HF treatment produced *ent*-calyculin A in 69% yield and *ent*-calyculin B in 84% yield.

#### 5.6.5. Total synthesis of calyculin C by Armstrong [21,65]

**Scheme 55:**
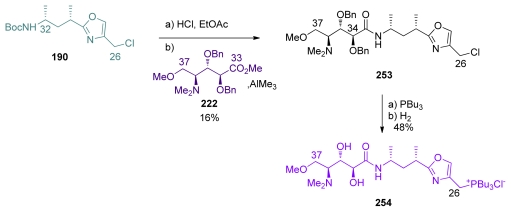
Preparation of C_26_–C_37_ fragment **254.**

The total synthesis of calyculin C by Armstrong was also a result of perseverant work. Armstrong and co-workers were also the first to prepare calyculin C and prove its stereochemistry. Boc-deprotection of **190** was followed by coupling with ester **222** (Scheme 55). Unfortunately, this reaction produced a 2.7:1 mixture of C_34_ epimers. Further studies proved that the main product was the undesired isomer; the expected diastereomer **253** being isolated in only 17%. Conversion of the chloride to the phosphonium salt and benzyl-deprotection then furnished **254**.

**Scheme 56:**
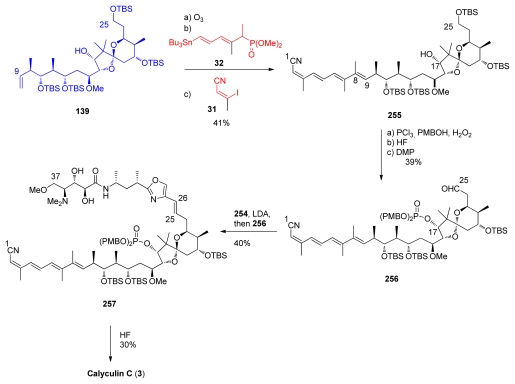
Final steps to calyculin C.

The final stages of the synthesis followed Evans’ example (Scheme 56). Fragment **139** was converted to the tetraene **255**. C_17_-phosphonate protection, C_25_-deprotection and subsequent oxidation furnished aldehyde **256**. Olefination between **256** and **254** furnished compound **257** in modest yield. Final HF treatment completed the total synthesis of calyculin C **3**.

#### 5.6.6. Formal total synthesis of *ent-*calyculin A by Barrett [23]

**Scheme 57:**
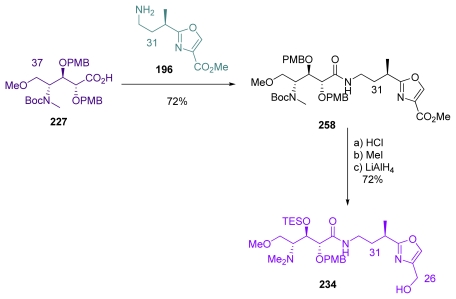
Preparation of the C_26_–C_37_ fragment **234** by Barrett.

Barrett *et al*. last steps for completing the total synthesis of *ent-***1** began with the coupling of aminoacid **227** and oxazole **196** fragments, to give amide **258** (Scheme 57). The latter was then converted to Evans’s intermediate **234**, by a three-step/one-pot sequence involving Boc-deprotection, *N*-methylation and reduction of the ester at C_26_.

**Scheme 58:**
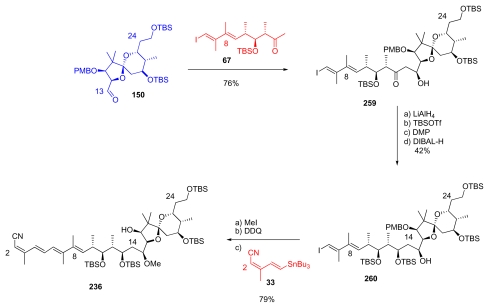
Preparation of the C_1_–C_25_ fragment **236** by Barrett.

Aldol reaction between methyl ketone **67** and aldehyde **150** gave in a diastereoselective manner β-hydroxyketone **259**, unfortunately carrying the wrong stereochemistry at C_15_ (Scheme 58). LiAlH_4_ reduction at C_13_ furnished the corresponding diol in 73%, together with its C_13_-epimer in 15% yield. Selective monosilylation at C_13_ was followed by inversion of stereochemistry at C_15_ by successive Dess-Martin oxidation and DIBAL-H reduction to produce **260**. *O*-Methylation at C_15_, PMB-removal at C_17_ and final Stille coupling with stannane **33** finished the synthesis of Evans’ intermediate **236**.

## 6. Conclusions

Altogether six total or formal synthesis of calyculins have been published. These syntheses can be divided into three groups:

- Masamune and Armstrong have described the total synthesis of natural calyculins A and C, respectively
- Evans and Smith have completed the total synthesis of the enantiomer of naturally occurring calyculin A (and B for Smith)
- Shioiri and Barrett have published highly advanced intermediates, previously prepared by Masamune and Evans respectively and therefore accomplished formal synthesis of natural and non natural calyculin A.

Even if the basic retrosynthetic analysis appeared to be quite similar in the different synthesis, the different points of views and methods makes the comparison challenging. The overall yields and number of steps of the different syntheses are compiled in Table 2.

Each of these syntheses resulted from extensive efforts and has to be considered as high level work. As discussed earlier, the structure of calyculins makes the total synthesis very demanding in every aspect; every fragment had its own features and presented challenges. Of course, theses syntheses are not perfect and some drawbacks could be noted, like a C_13_-stereochemistry inversion for Evans, a low-yielding iodide-tin exchange for Masamune, protecting groups exchange for Shioiri, low selectivity in the final Peterson olefination for Smith (which fortunately led to the formation of *ent*-calyculin B), stereochemistry inversions and poor selectivity in the second Brown crotylation for Armstrong or a C_15_-stereochemistry inversion for Barrett. However, all these syntheses were performed before 2000 and all the new tools that have appeared in the last decade for stereoselective transformations were not available. For all these reasons, these total syntheses of the structurally much elaborated calyculins deserve the biggest respect from the synthetic community.

Another goal should still lie in a better understanding of the PP inhibition-activity of calyculins and related toxins. Improved design and synthetic methods should also lead to the design of simpler synthetic inhibitors that could compete with the activity of natural toxins.

## Figures and Tables

**Scheme 6:**
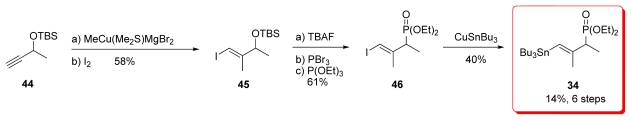
Preparation of phosphonate **34.**

**Scheme 11:**
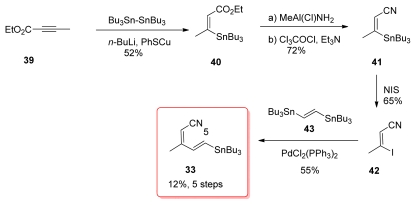
Preparation of stannane **33** by Barrett.

**Scheme 20:**
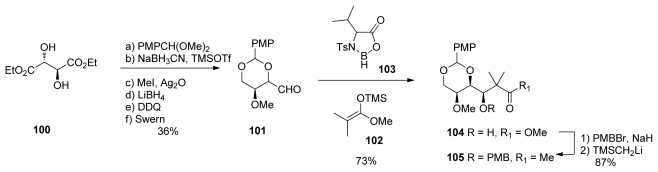
Preparation of ketone **105**.

**Scheme 24:**
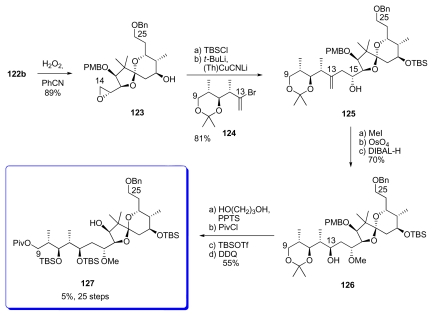
Preparation of fragment **127.**

**Table 1:** PP1 and PP2A inhibitors.

Name of the inhibitor	Isolation origin	Structural Scaffold	IC50 nM a		Properties	Ref.
			PP1	PP2A		
Microcystin-LR (**18**)	Blue green algae	Cyclic peptide	0.3–0.6	0.04–2.0	Liver toxin	[24]
Nodularin-V (**19**)	Blue green algae	Cyclic peptide	0.5–3	0.03–1.0	Liver toxin	[24]
Cantharidin (**15**)	Blister beetles	Terpenoid	0.5–2.0	0.2	Natural defensive toxicant	[6]
Thyrsiferyl- 23-acetate (**16**)	Red algae, *L. Obtusa*	Terpenoid	>1	(4–16)·10^−3^		[6]
Okadaic acid (**13**)	*Dinoflagellates*	Polyketide	10–1300	0.02–1.0	Tumour promoter	[24]
Dinophysistoxin-4 (**14**)	*Dinoflagellates*	Polyketide	~200	~2		[4]
Calyculin A (**1**)	*Marine sponge D. calyx.*	Polyketide	0.4–2.0	0.25–3	Tumour promoter	[24]
Calyculin C (**3**)	*Marine sponge D. calyx.*	Polyketide	0.6	2.8	Tumour promoter	[24]
Tautomycin (**20**)	Bacterium, *Streptomyces verticillatus*	Polyketide	1.1–7.51	10–23.1	Antibiotic	[24]
Fostriecins (**17**)	Bacterium, *Streptomyces pulveraceus*	Polyketide	0.131	3.4·10^−6^	Antitumoric activity	[4]

a The determined IC_50_ values are not always directly comparable from source to source. They may vary depending on the substrate, and on the purity, concentration and origin of the purified protein.

**Table 2:** Overview of the total synthesis.

Group	Target	Pub. year	Number of steps a	Overall yield (%) a	Number of steps b	Overall yield (%) b
Evans	*ent*-Calyculin A	1992	33	0.54	36	-
Masamune	Calyculin A	1994	43	0.31	45	0.18
Shioiri	Calyculin A	1996	32	0.092	32	0.092
Smith	*ent*-Calyculin A	1998	35	0.89	37	0.79
Armstrong	Calyculin C	1998	30	0.018	30	0.018
Barrett	*ent*-Calyculin A	2001	34	0.9	34	0.9

a Longest linear sequence based on the reported starting materials.

b Longest linear sequence based on commercially available starting materials.
